# Impact of Automation Level of Dairy Farms in Northern and Central Germany on Dairy Cattle Welfare

**DOI:** 10.3390/ani14243699

**Published:** 2024-12-21

**Authors:** Lianne Lavrijsen-Kromwijk, Susanne Demba, Ute Müller, Sandra Rose

**Affiliations:** 1 Department of Agricultural Machinery, University of Applied Sciences, 17033 Neubrandenburg, Germany; susanne.demba@gmx.de; 2 Institute of Animal Science, University of Bonn, 53113 Bonn, Germany; ute-mueller@uni-bonn.de; 3 Department of Agricultural Process Engineering, Faculty of Agricultural and Environmental Sciences, University of Rostock, 18051 Rostock, Germany

**Keywords:** automation, dairy cattle welfare, automatic milking system (AMS), automatic feeding system (AFS), Welfare Quality^®^ Assessment

## Abstract

An increasing number of automation technologies for dairy cattle farming, including automatic milking, feeding, manure removal and bedding, are now commercially available. The effects of these technologies on individual aspects of animal welfare have already been explored to some extent. However, as of now, there are no studies that analyze the impact of increasing farm automation through various combinations of these technologies. The objective of this study was to examine potential correlations between welfare indicators from the Welfare Quality^®^ Assessment protocol and dairy farms with varying degrees of automation. To achieve this, 32 trial farms in Northern and Central Germany were categorized into varying automation levels using a newly developed classification system. The Welfare Quality^®^ Assessment protocol was used to conduct welfare assessments on all participating farms. Using analysis of variance (ANOVA), overall welfare scores and individual measures from the protocol were compared across farms with differing automation levels. No significant differences were observed in overall welfare scores, suggesting that the impact of automation does not exceed other farm-related factors influencing animal wellbeing, such as housing environment or management methods. However, significant effects of milking, feeding, and bedding systems on the appropriate behavior of cattle were observed. Higher levels of automation had a positive impact on the human–animal relationship and led to positive emotional states. Moreover, farms with higher automation levels had significantly lower scores for the prevalence of severe lameness and dirtiness of lower legs. It could be concluded that a higher degree of automation could help to improve animal welfare on dairy farms.

## Simple Summary

Automation in dairy farming is becoming increasingly common in Germany, with most technologies focusing on labor-saving and providing relief from physically strenuous labor for farmers. However, the impact of increased automation on dairy cattle welfare has remained largely unexplored. Consequently, this study developed a classification system that enabled the division of 32 trial farms into different levels of automation and evaluated each farm’s welfare status using the international Welfare Quality^®^ Assessment Protocol. The findings suggested that increased levels of automation foster positive effects on animal behavior and human–animal relationships, as cows in highly automated farms are less disturbed and experience greater freedom. Additionally, the results showed that automation techniques can aid farmers in improving health indicators such as the prevalence of lameness and cow cleanliness.

## 1. Introduction

Farm processes and management in dairy cattle farming have always demanded significant human labor input. Economic factors, such as increasing labor costs, as well as farmers’ desire for less strenuous and repetitive work and more flexible labor conditions, have led to the development of automation systems designed to replace the most time-consuming activities on dairy farms [1]. In this context, automation is defined as the execution of a function by a machine that was initially performed by a human [2]. Work processes on dairy farms that have been automated in recent decades include milking, feeding, manure removal, cubicle bedding, and health monitoring. Different farms utilize varying extents of automation techniques, resulting in diverse levels of automation on dairy farms. The concept of automation levels has been previously described by Billings in reference to aviation [3]. These levels range from direct manual control to primarily autonomous operations where human involvement is minimal. This concept translates well to automation in other areas.

In the context of milking, conventional milking systems (CMS) remain a common technique for milk production in German dairy farms [4]. The development of automatic milking systems (AMS) began in the mid-1970s and was initially installed in commercial farms during the 1990s [5]. Currently, 50–70% of newly installed milking systems in Germany are AMS. In terms of the total number of dairy farms, 3% of farms in Germany used AMS for milking in 2011 [6]. Globally, more than 38,000 farms have incorporated AMS into their barns [7]. There is an intermediate form between conventional and automatic milking systems known as batch milking, where cows are herded to the waiting area two or three fixed times per day and then milked by AMS.

In Germany, dairy cattle are commonly fed using a tractor-drawn or self-propelled feed mixer wagon driven by a person. According to Haidn [8], automatic feeding can be divided into three stages: I. mixing–distribution; II. filling mixer–mixing–distribution; and III. unloading and transport–filling mixer–mixing–distribution.

Frequently pushing feed towards cows at the feeding table is crucial in dairy cattle feeding, often performed by the farmer using a tractor or wheel loader. Nowadays, this can also be accomplished using automatic feed pushers, that dispense feed regularly without any human labor according to the set schedule [9]. Moreover, dung removal needs to be managed in cubicle housing systems. This can be achieved on slatted floors without any technical assistance or human labor, or on solid floors using a tractor or wheel loader equipped with attached scrapers. Nowadays, automatic dung removal is facilitated by automatic manure scrapers or manure robots [10]. Lastly, the process of lining the cubicles with sufficient organic material can be performed manually by the farmer or with the help of a cubicle bedding dispenser. Two different technologies are available to automate this task: a rail-guided robot that moves above the cubicles distributing the bedding material (e.g., Astor, Hetwin Automation Systems GmbH, Langkampfen, Austria), or a pipe system that blows chopped straw into the cows’ cubicles at preset times (e.g., Strohmatic, Schauer Agrotronic GmbH, Prambachkirchen, Austria).

Most automation techniques in dairy farming prioritize farmer benefits, particularly the labor-economic aspects, by providing more flexibility and reducing manual labor. Time savings by AMS are often touted as their most significant benefit. Studies by Rodenburg [11] and Heikkila et al. [12] report a 29 to 30% time saving on farms using AMS. In contrast, Butler et al. [13] suggest that while AMS provides farmers with more labor flexibility, it does not necessarily reduce labor time. Instead, physical tasks like feeding or milking in automated systems are supplanted by management tasks such as data consultation, alarm monitoring, and maintenance and repair of digital tools and techniques [14]. As a result, managing digitized farms necessitates new farmer skill sets, including technological literacy and data management. This requirement can heighten farmers’ demands [15], potentially increasing their mental workload [1]. Some farmers, however, feel less stressed, as digital tools assist in identifying cows in heat or with physiological problems, which are typically challenging to spot visually [16]. Interviews by Goller et al.’s [15] interviews discovered that farmers generally perceive the implementation of digital technologies positively, as it allows more work flexibility and improves work–life balance. A survey conducted on Canadian AMS farms about farmers’ mental health found that those utilizing automatic feeding systems (AFSs) experienced less anxiety, depression, and stress and displayed greater resilience than those feeding manually [17]. Besides the effects of automation technologies on farmers’ lives, it is also crucial to examine the impact of increased automation in dairy farming on animal welfare.

Animal welfare pertains to the physical and mental state of an animal concerning its living conditions. It is deemed optimal when the animals are healthy, comfortable, well-nourished, safe, and free from unpleasant states like pain, fear, and distress. The ability to express behaviors important to their physical and mental wellbeing is also an essential component of animal welfare [18]. Numerous evaluation systems have been developed over the past decades to assess animal welfare on dairy farms, each comprising different combinations of indicators tailored to various interests, such as dairy farm government controls, food product certification/labeling, self-monitoring, or consultation, policy, and research. In Germany, the commonly used welfare assessment tools for dairy cattle include the printed guideline “Tierschutzindikatoren: Leitfaden für die Praxis—Rind” [19] and the digital application “CowsAndMore” [20]. More globally, numerous other welfare assessment systems exist, with the “Farmers Assuring Responsible Management (FARM) Animal Care Program” from the United States, the “Code of Welfare: Dairy Cattle” from New Zealand, or “Welzijnswijzer Melkvee” and “KoeKompass” from the Netherlands serving as some examples [21,22]. The most widely recognized and extensive assessment tool is likely the Welfare Quality^®^ Assessment protocol [23], developed and tested by senior scientists from 20 European countries [21]. The protocol, intended for use by third parties such as certification and inspection bodies, enforcement agencies, advisory bodies, and research groups, was tested on 91 farms across ten different countries [24]. It rests on four main welfare principles—Good Feeding, Good Housing, Good Health, and Appropriate Behavior—which are then divided into 12 welfare criteria, further including 27 welfare measures for dairy cows. These measures are rated on a scale from 0–100. The scores for the welfare measures then determine the scores for the welfare criteria, which are scored on the same scale and subsequently converted into principle scores. Ultimately, these principle scores are transformed into a final overall assessment score for farms [21].

Contrary to other welfare assessment protocols, the Welfare Quality^®^ Assessment protocol includes more animal-based measures than resource- and management-based ones [25,26]. While housing and management conditions are acknowledged as significant factors influencing the quality of animals’ lives, Blokhuis [27] views animal-related measures as more crucial due to the often nebulous connection between housing and welfare. Accordingly, Welfare Quality^®^ focuses on evaluating animals’ mental and physical wellbeing rather than their living conditions. Only when valid animal-based measures are not available do management- and resource-based measures come into use for assessment [26]. By incorporating 60% animal-based measures, the protocol can illustrate extensive variations between farms, rendering it applicable to many different types of farming systems [24,28]. Furthermore, the Welfare Quality^®^ Assessment is well-suited for research due to its use of a protocol with standardized descriptions and calculation methods for scoring; assessments can be completed in a reasonable amount of time; there is no special equipment or profession required, and it yields a quantifiable output, making it possible to measure differences between farms [24,27].

Several researchers have already utilized the Welfare Quality^®^ Assessment protocol for varied research purposes. For instance, Wagner et al. [29,30] explored the effects of daily grazing times on dairy cattle welfare in organic and conventional farms, as well as comparing differences in animal welfare between organic and conventional farms in general. Popescu et al. [31,32] investigated the effects of housing systems (loose vs. tie-stall; with regular outdoor exercise vs. without access to exercise in tie-stalls) on the welfare of dairy cows by using the Welfare Quality^®^ Assessment protocol, while Gieseke et al. [33] used it to assess the effects of cubicle characteristics on dairy cattle welfare.

Furthermore, the impact of individual automation techniques on specific aspects of animal welfare has already been investigated. Numerous studies have considered automatic milking. One significant benefit of AMS for the animal appears to be the individualized milking frequencies for cows, which can fluctuate based on different production levels and lactation stages [34]. Particularly during times of personnel shortage and fluctuation, the consistency of the milking process offered by AMS can be seen as beneficial for animal welfare [34]. This consistency, as well as more frequent milkings, may lead to improved udder health. Differences in udder health and somatic cell counts between CMS and AMS have been examined in several studies. The results of these studies have varied widely, ranging from no changes in udder health [35,36] to deteriorations [37,38,39] and improvements [40].

Furthermore, studies examining differences in cow behavior between CMS and AMS are available. These studies explored milk and plasma cortisol concentrations, heart rates, heart rate variability, and step-kick behavior during milking. No significant differences were observed between the two milking systems [41,42,43]. There were concerns that less direct contact between farmers and their animals in AMS farms could negatively impact the human–animal relationship. Contrarily, Wildridge et al. [44] demonstrated otherwise. Their study indicated that, despite farmers spending less time with their cows, the animals were less anxious around humans. This was based on shorter avoidance distances and lower stress responses to close contact. Gaworski and Kic [45] suggested that AMS is perceived as less stressful compared to CMS. This is because cows in CMS are frequently interrupted in their lying areas when they are forced to rise for milking. Additionally, cows milked conventionally may experience stress in the waiting area before milking, where they are gathered for extended durations, which curtails their ability to exhibit natural behaviors.

The welfare of dairy cattle can also be impacted by automatic feeding. Grothmann et al. [46] reported that higher feeding frequencies led to significantly higher feed intakes but they did not identify any alterations in feeding behavior and rumination. Oberschätzl-Kopp et al. [47] found that a feeding frequency of six times per day, compared to twice per day, resulted in a more uniform distribution of cows at the feed bunk, shorter waiting times to access the feed bunk, and reduced disturbances during feeding. The authors considered these findings to be an improvement in animal wellbeing. Even higher feeding frequencies could lead to a decrease in long-duration lying bouts, due to the continual disruption of cows’ resting times [48]. The ability to program feeding frequencies, times, and doses for feeding through automatic feeding systems, thereby enabling more precise control over feeding, is seen as beneficial for both management and animal welfare. Automatic feeding systems also allow for the feeding of specialized rations to different performance groups in smaller herds. Increased feeding frequencies can assist in optimizing the dry matter intake of cows, leading to improved rumen pH stability. Moreover, distributing feed more often decreases the time that fresh feed remains on the feeding table, reducing the risk of contamination and aberrant feed fermentation [49,50]. Gaworski and Kic [45] also viewed the electric motors of feeding robots as a benefit due to the reduction in noise exposure and emissions in barns.

Concerning automated dung removal, it could present a lesser incidence of lameness and claw diseases, thereby positively impacting animal welfare. Studies by Doerfler et al. [51] and King et al. [52] suggested that the use of robotic scrapers led to reduced infectious claw diseases and less prevalence of clinical lameness. Additionally, they noted a significant decrease in greenhouse gas emissions. An optimized dung removal frequency could lessen the soiling levels on dairy cattle walkways, thus minimizing the risk of animals slipping. Particularly, robotic scrapers equipped with a water spray function may prevent the accumulation of smear layers, further reducing instances of animal slipping [53]. Considering the effect of robotic walkway cleaning on clinical mastitis and somatic cell counts, Doerfler et al. [54] observed a decreased incidence of clinical mastitis after the implementation of a robotic scraper, while somatic cell counts simultaneously increased. Besides the health-related benefits of robotic scrapers, the behavioral response to this technology by the animals also merits consideration. Accordingly, research has been conducted, including the analysis of cows’ cardiac activity and the measurement of stress responses through the concentration of cortisol metabolites in fecal samples. Animal behavior was also observed to evaluate their reactions to robotic scrapers. According to Buck et al. [55] animals showed lower cardiac activity while the scrapers were operational and their feeding behavior could be affected by the scrapers. Doerfler et al. [56] found a decline in heart rate variability and a constant cortisol metabolites concentration after the introduction of a robot scraper. However, despite these minor disruptions, studies have also shown that cows adjust well to scrapers [57].

At this stage of knowledge, studies examining the effect of automatic bedding systems on dairy cattle welfare are limited. It is conceivable that automatic bedding systems could enhance the quality of cubicles in terms of the abundance of bedding material and cleanliness. A consistent abundance of bedding material in cubicles might result in greater lying comfort, potentially leading to longer periods of rest for the cows [58,59]. This, in turn, could ease the strain on claws and joints, reduce the incidence of lameness, and increase milk production [17,60,61].

Cleaner cubicles can also lead to cleaner cows, particularly with regard to udder cleanliness, and could potentially improve udder health [62].

Many studies have already investigated the impact of individual automation technologies on single aspects of animal welfare. The aim of this study, however, is to more holistically analyze the influence of different combinations of automation technologies on cow welfare. It is hypothesized that a higher degree of automation in dairy farms results in increasing animal welfare through a better assessment of animal welfare traits. To this end, farms with varying degrees of automation technologies were classified into different automation levels. These levels were then compared in terms of animal welfare using the Welfare Quality^®^ Assessment protocol [63].

## 2. Materials and Methods

### 2.1. Farms and Animals

The study was carried out from October 2023 to April 2024. Data were collected from 32 German dairy farms located in both Northern and Central Germany. The inclusion criteria for the farms were based on specific conditions: possession of Holstein-Friesian breed cows, absence of access to pasture, practice of conventional farming (excluding organic farms), and accomodation in cubicle housing. Table 1 presents the herd sizes of the farms following Gargiulo et al. [64] and the yielded 305-day milk production data.

### 2.2. Classification of Farms According to Automation Level

Based on our current knowledge, there is no existing system for classifying dairy farms into different levels of automation. As a result, we developed our own classification system.

All farms in this study employ various automation techniques in diverse combinations. To construct a classification system for automation, scores were allocated across four functional areas: milking, feeding, dung removal, and bedding (Table 2). The less human labor required by the implementation of a specific technique, and the lesser the disturbance caused to the animals by it, the higher the number of points awarded [3,65].

Thus, traditional methods for the specified functional areas receive zero points. Work completed using the highest level of available technology is awarded two points. This double-point award, rather than just a single point, is designed to allow for a gradation of partial automation in the areas of milking and feeding, where batch milking and automatic feed pushing represent partial automation of the functional area.

In this paper, conventional feeding is defined as the process carried out entirely by human labor, using such tools as tractors, wheel loaders, and feed mixers. On the other hand, automatic feeding is classified as stage 2 of automatic feeding, as outlined by Haidn [8]. This is because there are currently no commercial techniques available for stage 3.

The scoring system enables a gradation from no automation to highly automated dairy farms, with a range of scores from zero to eight: zero represents the least automated and eight the most automated dairy farms. This is comparable to the “levels of mechanization” by Kern and Schumann [66], who devised a classification system comprised of three areas: pre-mechanization, mechanization, and automation. These areas can be further divided into nine levels, spanning from manual to automatic manufacturing (Table 3).

Automation scores were computed for all farms by summing up the points assigned to each functional area. These points ranged from 0 for conventional processes to 1 for intermediate stages in milking and feeding areas, and 2 for fully automated processes. This resulted in potential total scores ranging from zero to eight for each farm. For instance, farms without automation in any functional area received a score of zero, while those with automation in all functions attained the highest possible score of eight points. Farms with varied levels of automation could receive scores ranging from 1 to 7, depending on the combination of automation processes (Table S1).

The distribution of automation scores for all farms is summarized in Table 4. Furthermore, these automation scores were classified into automation levels based on Kern and Schumann [66].

### 2.3. Welfare Assessment

Cow welfare was documented on all farms using the Welfare Quality^®^ Assessment protocol [63], which includes 27 measures for dairy cattle (Table 5). All the data were gathered by a single individual, who spent 1 to 2 days on each farm. This person had previous experience in dairy farming and handling as well as in welfare assessment. Animal welfare assessment was taught as a part of a lecture module with a total of 32 units of 45 min each at the Neubrandenburg University of Applied Sciences. Each farm was visited only once for data sampling. All farms were evaluated by the same person, who proceeded as described in the chapter 6.1 of the Welfare Quality^®^ Assessment protocol on each farm. The sample size per farm depended on farm size and was determined according to the guideline “Tierschutzindikatoren: Leitfaden für die Praxis—Rind” as the requirements in the protocol only cover a herd size of up to 300 animals. Management-related measures were collected by interviewing farm managers before or after the assessments. Farm managers also supplied information about milk somatic cell count, mortality, and dystocia via milk recording reports and data from their herd management software data. The “percentage of downer cows” as well as “expression of other behaviors” were excluded from data analysis due to the absence of valid records for these indicators on the farms. The assessor documented both resource-based measures and animal-based indicators in the barn as outlined in the Welfare Quality^®^ Assessment protocol [63]. Measures were grouped into criterion values ranging from 0 to 100 with ‘0’ representing the worst situation, ‘50’ a neutral situation, and ‘100’ the best situation where further welfare improvements cannot be considered. These values are then allocated into principle scores, also ranging from 0 to 100, which are the foundation for the calculation of overall scores (excellent, enhanced, acceptable, not classified) [63]. The collected welfare measures were processed into criteria, principles, and overall scores using the protocol’s online software program.

### 2.4. Statistical Analysis

Statistical analyses were conducted using SPSS 29 (IBM, Armonk, NY, USA). Parameters were evaluated at the farm level and tested for normal distribution with the Shapiro–Wilk Test. Levene’s test was employed to test the homogeneity of variance. Initially, Welfare Quality^®^ principles, criteria, and measures were analyzed to compare differences between farms with various levels of automation. Data with a normal distribution were analyzed with ANOVA, while data not normally distributed were tested with the Kruskal–Wallis Test. Various covariates were incorporated for individual indicators in the ANOVA computations. Since these covariates did not significantly impact the results, they were removed from the statistical model. Secondly, the effects of specific automation techniques (such as milking, feeding, bedding, and dung removal systems) were analyzed on distinct welfare principles considered to directly relate to others (e.g., feeding system and good feeding). Here, normally distributed data were tested using Student’s t-test or ANOVA; in contrast, not normally distributed data were tested with the Mann–Whitney U test or Kruskal–Wallis test, depending on whether there were two or more groups to compare.

All data are presented as means, along with minimum and maximum values. A significance level was established at *p* ≤ 0.05. For significant values, a post hoc multiple comparison was conducted, using the Bonferroni method, to ascertain the extent of the differences between the groups.

As the data for “ease of movement”, “absence of pain induced by management procedures”, and “expression of other behaviors” were identical across all farms, they were excluded from statistical analyses. The identical “ease of movement” scores are attributed to all farms using loose housing systems, resulting in a maximum score of 100. Each farm achieved a uniform score of 75 for “absence of pain induced by management procedures” due to their shared management practices—the absence of tail docking and the application of thermal dehorning with the use of analgesics and anesthetics. Lastly, none of the farms attained any points for “expression of other behaviors” as no farms provided their cows access to pasture.

## 3. Results

For overall welfare scores 90.6% of farms were rated as “enhanced” according to the Welfare guidelines from 2009 [63]. There were three farms, accounting for 9.4%, which were graded as “acceptable”. Among these three, two were non-automated farms, and one was highly automated. Notably, none of the trial farms received a rating of “not classified” or “excellent”. It should be noted that no trial farm could achieve the “excellent” score as none of the farms provided cows with access to pasture. This lack of pasture access resulted in lower scores for the “appropriate behavior” welfare principle; thus, an “excellent” score was unattainable.

The small differences between overall welfare scores necessitate a more detailed examination of differences among the Welfare Quality^®^ criteria and measures.

As shown in Table 6, significant differences can be observed between farms of varying automation levels concerning the “appropriate behavior” of animals (*p* < 0.001). This is seen specifically in aspects such as “good human–animal relationship” (*p* = 0.015) and “positive emotional state” (*p* < 0.001). Farms with higher levels of automation demonstrate significantly higher scores for these criteria.

A pairwise comparison of appropriate behavior, good human–animal relationship, and positive emotional state reveals that the significant differences mainly arise from the comparison between non-automated and fully automated farms (Table 7).

Significant differences were found in the single Welfare Quality^®^ measures for “percentage of cows with dirty lower legs” (*p* = 0.049) under the good housing principle and “percentage of severely lame cows” (*p* = 0.03) under the good health principle. Both criteria showed lower values on farms with higher levels of automation.

Significantly lower values were found on farms with higher automation levels for the principle of “appropriate behavior”, specifically for the “percentage of cows that can be approached between 50 and 100 cm” (*p* = 0.013) and the “percentage of cows with an avoidance distance greater than 100 cm” (*p* = 0.005). This indicates that as farm automation increases, the percentage of cows showing greater avoidance distance decreases.

For the remaining Welfare Quality^®^ measures, no significant differences could be found between groups of farms.

The effects of individual, specific automation techniques on varying Welfare Quality^®^ principles are analyzed in Table 8. The type of bedding system had a significant impact on the “good housing” principle (*p* = 0.014), most notably affecting the cleanliness of cows, as evidenced by the “percentage of cows with dirty udders and dirty lower legs” (*p* = 0.005; *p* ≤ 0.001). In this case, farms with a greater degree of automation achieved significantly lower scores for dirtiness of udders and lower legs.

The dung removal system did not affect “good housing”.

“Good health” showed no significant correlations between the different automation techniques. Farms with automation technologies in milking, feeding, and bedding demonstrated significantly higher scores regarding the “appropriate behavior” of cows.

## 4. Discussion

A total of 32 dairy farms were classified into three distinct levels of automation. Considering the overall scores from the Welfare Quality^®^ Assessment protocol, no significant differences were observed between highly automated farms and those without automation. Nearly all trial farms (n = 29) received a welfare rating of “enhanced”, showing a uniform welfare status. The remaining three farms were deemed “acceptable”, including two non-automated farms and one highly automated farm. It is important to consider that many welfare indicators incorporated in the Welfare Quality^®^ Assessment protocol for cattle are not directly related to automation. This includes factors such as water provision or farm cubicle design. Therefore, it is crucial to analyze particular welfare criteria and measures associated with automation techniques in dairy cattle farms.

The results of this study showed that farms with increasing levels of automation achieve significantly higher scores in appropriate behavior and good human–animal relationship, as well as positive emotional state.

This aligns with previous finding by Holloway et al. [67] and Driessen et al. [68]. These researchers reported that farmers described their herds as more “relaxed”, “happy”, “quiet”, “cool”, and “chilled out” after switching to an automatic milking system, particularly when farmers or other people move among the cows. Furthermore, they suggested that cows, given the choice of when to be milked and the ability to follow their own routines, can exhibit individual behaviors. This increased freedom aligns with our findings of higher scores of positive emotional states on farms with higher levels of automation. Jacobs and Siegford [69] also maintain that cows milked by AMS have more freedom to establish their own daily routines, giving them more opportunities to interact with their environment and supporting the natural behavior of cows. Gaworksi [9] points out that another aspect of AMS is that it offers more freedom to cattle and does not interrupt their resting periods, as opposed to CMS, where cows are disturbed 2–3 times a day to go to the milking parlor. Additionally, AMS eliminates waiting times in collecting yards, where cows may experience discomfort standing in crowded areas with limited space, inhibiting their natural behaviors [9].

Our finding of improved human–animal relationships in farms with increased automation levels aligns with the findings of Wildridge et al. [44], who also reported enhancements in human–animal relationships on automated farms. Their study investigated changes in farmer routines, human–animal interactions, and cows’ avoidance distance, concluding that while farmer–cow interaction times decreased, both avoidance distance and cattle stress responses improved. This suggests that transitioning to AMS fosters an improved human–animal relationship, with cows appearing calmer and less fearful of humans. Despite the decrease in interaction times, the improvement in relationships may be attributed to the change in the nature of contact between farmers and animals. Instead of herding cows to the milking parlor at fixed times each day, farmers now slowly navigate through the herds to check animals and machinery, or to bring forward cows, who have not visited the robot for an extended period [44].

Furthermore, results showed an impact of the feeding system on appropriate behavior, with higher values for farms that had higher automation levels. However, this is very dependent on the number of feeding times per day. Numerous studies have investigated the impact of feeding frequencies and feed pushing per day on the feeding and resting behavior of cows. For example, De Vries et al. [49] and Da Borso et al. [50] conducted experiments that showed a change in feeding time distribution to a higher frequency resulting in a more even distribution of cows at the feeding table throughout the day. Nevertheless, the feeding frequency did not affect the cows’ lying times or the amount of agonistic interactions at the feeding table. Additionally, Mattachini et al. [70] confirmed a more even distribution at the feeding table throughout the day when cows were fed eleven times per day, as opposed to six times.

De Vries et al.’s study [49] reported that feeding frequency does not affect the daily incidence of aggressive interactions, a finding in line with our results. We found no significant differences in agonistic interactions (frequency of head butts and displacements per hour) between farms of differing automation levels. Conversely, Phillips et al. [71] discovered that increasing feeding frequencies resulted in fewer cows being displaced at the feeding table.

In contrast to the findings of De Vries et al. [49] and Mattachini et al. [70], Grothmann et al. [46] and Mattachini et al. [48] observed that higher feeding frequencies led to a reduction in time spent lying down. This could have a negative impact on the resting behavior of dairy cows.

While studies regarding cows’ response to automatic bedding systems are not available at this time, their behavior towards automatic scrapers has been investigated by Buck et al. [55]. Their study suggests that in certain situations, such as during feeding or when in crowded areas, cows perceive manure scrapers negatively. However, these effects appear to be minimal, leading to the conclusion that animals can adapt to the use of scrapers quite easily. This conclusion aligns with the findings of studies conducted by Stülpner et al. [56] and Doerfler et al. [57].

With regards to the cleanliness of cows and lameness, it was found that farms with the highest level of automation had the smallest proportion of cows with dirty lower legs, and no severely lame cows at all. Automation level 2 is the only category including farms with automatic bedding, which is likely the reason for improved claw health and cow cleanliness. This is backed by van Eerdenburg and Ruud [72], who claim that housing comfort is heavily influenced by the amount and quality of bedding. The impact of the bedding system on the cleanliness of cows was also proven to be significant by comparing the Welfare Quality^®^ principle “good housing” with the bedding system, which showed a significant effect. Farms with automatic bedding systems litter their cubicles at least once every day. This results in drier, cleaner, and softer cubicles which are preferred by cows, as stated by Schütz et al. [73]. This leads to improvements in welfare in terms of the quantity and quality of lying times [74,75,76]. Furthermore, Schütz et al. [73] found that deep bedding, when compared to rubber mats, reduces the number of cows with dirty legs and decreases lesions and swellings.

Concerning the proportion of lameness in herds of dairy cattle herds, King et al. [52] observed that lameness is less prevalent with frequent dung removal frequencies in manure alleys. This aligns with our findings; highly automated farms with no severely lame cows scraped their alleys at least once an hour, and 58% of these highly automated farms scraped alleys twice per hour. In contrast, non-automated farms had dung removal frequencies ranging from zero to twice per day. Similarly, Doerfler et al. [51] provided evidence of improved claw health following the implementation of robotic walkway scrapers. Obviously, regular and professional claw care is a major factor influencing lameness. Nonetheless, Freigang et al. [77] found that the quality of lying and walking areas also significantly impacted the lameness of dairy cattle.

The high variance of experimental farms, ranging from housing conditions to management and personnel, makes it challenging to analyze the effect of automation levels on the welfare of dairy cattle. An expansion in the number of trial farms would be ideal to acquire more substantial findings. The sample size of 32 experimental farms in this study was limited because no more than 12 highly automated farms could be located in Northern and Central Germany, as feeding and bedding robots are still not prevalent in these areas. Furthermore, there was an intention to standardize farms in terms of breed and conventional farming, which also restricted the selection of farms. An increasing number of dairy farms implementing automation technologies in the future would provide valuable data to further understand the impact of automation on cow welfare.

## 5. Conclusions

To analyze the impact of various combinations of automation technologies on cow welfare, a novel classification system was created to categorize dairy farms into different automation levels. This classification system was well-suited for the aim of this study and can be utilized for further research related to automation in dairy farming. Moreover, the results of this study showed significantly higher values for appropriate behavior, human–animal interactions, and positive emotional state for animals on farms of with higher automation, suggesting that automation in dairy farming has a significant influence on animal behavior. Also, the study results suggest that automation can assist farmers in improving housing and herd health using techniques such as automatic bedding and increased dung removal frequencies, which lead to improved cleanliness of cattle and a reduction in the incidence of severe lameness. Clean and dry cubicles and walking alleys could help to prevent mastitis as well. Detecting the somatic cell score by an AMS with each milking can enable early detection of udder infections.

## Figures and Tables

**Table 1:** Herd sizes and 305-day milk yield of trial farms according to Gargiulo et al. [64].

Herd Size	305-Day Milk Yield (Mean + SE) in kg
Small (<150 cows; n = 5)	11,569.00 ± 1913.95
Medium (151 to 300 cows; n = 8)	12,284.14 ± 1180.57
Large (301 to 500 cows; n = 6)	11,113.50 ± 499.11
X-Large (501 to 700 cows; n = 7)	10,951.29 ± 891.35
XX-Large (>701 cows; n = 6)	12,125.17 ± 868.15

**Table 2:** Automation scores of functional areas refer to Billings [3].

	0 Points	1 Point	2 Points
Milking system	Conventional milking system	Batchmilking	Automatic milking system
Feeding system	Conventional feeding system	Conventional feeding system with automatic feed pusher	Automatic feeding system
Dung removal system	Slatted floors or dung removal using a wheel loader or tractor	-	Manure scraper or manure robot
Bedding system	by hand or using a cubicle bedding dispenser	-	Bedding robot

**Table 3:** Levels of mechanization by Schumann and Kern [66].

Pre-mechanization	Manual
	Line flow
Mechanization	Single units with manual work
	Single units with mechanical control
	Multi-functional units without manual control
	Systems of units
Automation	Partly automated single units
	Partly automated systems
	Automated manufacturing

**Table 4:** Distribution of automation scores and allocation into higher automation levels.

Automation Level	Number of Farms	Automation Score	Number of Farms
0—no automation	10	0	3
		1	1
		2	6
1—semi-automation	10	3	1
		4	4
		5	5
2—high automation	12	6	6
		7	4
		8	2

**Table 5:** Principles, criteria, and measures of the Welfare Quality^®^ Assessment protocol for dairy cows version 3.2 published in February 2024 [63].

Principles	Criteria	Measures
Good feeding	1. Absence of prolonged hunger	Body condition score
	2. Absence of prolonged thirst	Water provision (number/length in cm of water troughs, bowls), cleanliness of water points, water flow, functioning of water points
Good housing	3. Comfort around resting	Time needed to lie down, animals colliding with housing equipment while lying down, animals lying partly or completely outside the lying area, cleanliness of udders, flank/upper legs, and lower legs
	4. Thermal comfort	As yet, no measure has been developed
	5. Ease of movement	Presence of tethering, access to outdoor loafing area or pasture
Good health	6. Absence of injuries	Lameness, integument alterations
	7. Absence of disease	Coughing, nasal discharge, ocular discharge, hampered respiration, diarrhea, vulvar discharge, milk somatic cell count, mortality, dystocia, downer cows
	8. Absence of pain induced by management procedure	Disbudding/dehorning, tail docking
Appropriate behavior	9. Expression of social behaviors	Agonistic behaviors (head butts, displacements)
	10. Expression of other behaviors	Access to pasture
	11. Good human–animal relationship	Avoidance distance
	12. Positive emotional state	Qualitative behavior assessment (defined by 20 terms of body language)

**Table 6:** Assessment of dairy cattle welfare according to automation levels (not automated vs. semi-automated vs. highly automated), presented as Welfare Quality^®^ principles and criteria in mean values (min-max), and significances of the group differences. *p*-values smaller than 0.05 are marked in bold (*p* < 0.05).

Welfare Quality^®^ Principles and Criteria	All Farms (n = 32)	Not Automated Farms (n = 10)	Semi-Automated Farms (n = 10)	Highly Automated Farms (n = 12)	*p*-Value
Good feeding	86.7 (24.2–100)	85.7 (52.9–100)	86.9 (60.9–100)	87.4 (24.2–100)	0.978
1. Absence of prolonged hunger	89.5 (46.5–100)	92.3 (66.4–100)	82.2 (46.5–100)	93.2 (49.6–100)	0.252
2. Absence of prolonged thirst	93.0 (13.8–100)	88.4 (46.5–100)	99.5 (95.0–100)	91.4 (13.8–100)	0.148
Good housing	78.6 (47.4–100)	74.4 (47.4–100)	74.1 (53.9–100)	85.9 (65.4–100)	0.132
3. Comfort around resting	66.1 (16.4–100)	59.3 (16.4–100)	58.9 (26.8–100)	77.7 (45.1–100)	0.132
4. Thermal comfort					
5. Ease of movement	100.0 (100–100)	100.0 (100–100)	100.0 (100–100)	100.0 (100–100)	-
Good health	40.9 (25.8–58.4)	39.2 (25.8–58.4)	41.8 (26.1–56.4)	41.6 (29.9–56.2)	0.790
6. Absence of injuries	79.6 (38.3–98.4)	78.9 (41.7–94.6)	73.0 (38.3–91.4)	85.8 (73.8–98.4)	0.133
7. Absence of disease	29.7 (11.5–51.4)	27.4 (11.5–51.4)	32.2 (12.9–48.6)	29.5 (14.6–48.8)	0.658
8. Absence of pain induced by management procedures	75.0 (75–75)	75.0 (75–75)	75.0 (75–75)	75.0 (75–75)	-
Appropriate behavior	33.5 (22.5–43.5)	29.3 (24.1–35.0)	31.6 (22.5–38.7)	38.5 (32.1–43.5)	**<0.001**
9. Expression of social behavior	98.5 (93.3–99.8)	98.0 (93.3–99.2)	98.7 (97.1–99.8)	98.8 (97.3–99.4)	0.258
10. Expression of other behavior	not applicable	not applicable	not applicable	not applicable	-
11. Good human–animal relationship	67.8 (29.8–95.6)	56.9 (42.2–74.4)	70.6 (29.8–93.8)	74.6 (51.2–96.0)	**0.015**
12. Positive emotional state	57.3 (33.2–79.2)	48.9 (33.2–66.1)	51.1 (33.4–67.8)	69.4 (59.9–79.2)	**<0.001**

**Table 7:** Bonferroni test for pairwise multiple comparisons of appropriate behavior, good human–animal relationship, and positive emotional state. *p*-values smaller than 0.05 are marked in bold (*p* < 0.05).

Welfare Quality^®^ Principles and Criteria	Automation Level I	Automation Level II	*p*-Value
Appropriate behavior	Not automated	Partly automated	0.636
		Highly automated	**<0.001**
	Highly automated	Not automated	**<0.001**
		Partly automated	**<0.001**
Good human–animal relationship	Not automated	Partly automated	0.099
		Highly automated	**0.016**
	Highly automated	Not automated	**0.016**
		Partly automated	1.0
Positive emotional state	Not automated	Partly automated	1.0
		Highly automated	**<0.001**
	Highly automated	Not automated	**<0.001**
		Partly automated	**<0.001**

**Table 8:** Differences between individual automation systems on Welfare principles. *p*-values smaller than 0.05 are marked in bold (*p* < 0.05).

Dependent Variable	Fixed Factor	All Farms	Automation Score 0	Automation Score 1	Automation Score 2	Sig.
**Good feeding**	Feeding system	86.7 (24.2–100)	79.8 (52.9–100)	89.4 (60.9–100)	88.7 (24.2–100)	0.239
Absence of prolonged hunger	Feeding system	89.5 (46.5–100)	81.7 (46.5–100)	85.5 (46.5–100)	97.6 (88.2–100)	0.077
**Good housing**	Bedding system	78.6 (47.4–100)	75.5 (47.4–100)	-	93.8 (82.8–100)	**0.014**
Percentage of cows with dirty udder	Bedding system	14.0 (0.0–50.5)	15.4 (0.0–50.5)	-	2.7 (0.0–8.0)	**0.005**
Percentage of cows with dirty flank/upper legs	Bedding system	25.0 (3.3–85.6)	26.5 (3.3–85.6)	-	10.7 (3.6–17.6)	0.117
Percentage of cows with dirty lower legs	Bedding system	33.7 (1.2–97.8)	38.2 (3.3–97.8)	-	5.0 (1.2–13.3)	**<0.001**
**Good housing**	Dung removal system	78.6 (47.4–100)	84.4 (59.1–100)	-	76.7 (47.4–100)	0.255
**Good health**	Milking system	40.9 (25.8–58.4)	40.8 (25.8–58.4)	33	41.4 (26.1–56.2)	0.683
Percentage of cows with milk somatic cell count of 400,000 or above	Milking system	14.1 (7.5–21.5)	13.2 (8.5–21.3)	19	14.4 (7.5–21.5)	0.457
**Good health**	Feeding system	40.9 (25.8–58.4)	39.2 (26.1–58.4)	40.4 (31.5–50.3)	42.4 (25.8–56.4)	0.736
**Good health**	Bedding system	40.9 (25.8–58.4)	40.6 (25.8–58.4)	-	41.6 (33.0–56.2)	0.819
Percentage of cows with milk somatic cell count of 400,000 or above	Bedding system	14.1 (7.5–21.5)	13.9 (7.5–21.5)	-	15.0 (7.7–19.0)	0.300
**Good health**	Dung removal system	40.9 (25.8–58.4)	37.9 (25.8–50.3)	-	41.9 (26.1–58.4)	0.285
Percentage of moderately lame cows	Dung removal system	6.7 (0.0–38.0)	5.4 (0.0–10.0)	-	7.2 (0.0–38.0)	0.915
Percentage of severely lame cows	Dung removal system	0.6 (0.0–4.4)	0.4 (0.0–1.3)	-	0.7 (0.0–4.4)	0.848
**Appropriate behavior**	Milking system	33.5 (22.5–43.5)	29.6 (24.1–35)	38.4	35.4 (22.5–43.5)	**0.021**
**Appropriate behavior**	Feeding system	33.5 (22.5–43.5)	29.1 (24.1–35)	33.3 (22.5–40.4)	36.3 (27.6–43.5)	**0.013**
**Appropriate behavior**	Bedding system	33.5 (22.5–43.5)	31.6 (22.5–38.8)	-	39.7 (37.6–42.2)	**<0.001**
**Appropriate behavior**	Dung removal system	33.5 (22.5–43.5)	31.7 (27.6–38.7)	-	34.1 (22.5–43.5)	0.307

## Data Availability

The dataset is available on request from the authors.

## Supplementary Materials

The following supporting information can be downloaded at: https://www.mdpi.com/article/10.3390/ani14243699/s1, Table S1: Calculation of automation scores for 32 trial farms.
