# Supplementary material for: Impact of Automation Level of Dairy Farms in Northern and Central Germany on Dairy Cattle Welfare
Source: Animals (Basel). 2024 Dec 21;14(24):3699. doi: 10.3390/ani14243699 (PMC11672561; doi:10.3390/ani14243699)
Supplement: Supplementary file 1 [file animals-14-03699-s001.zip › animals-3369322-supplementary.pdf]

Table S1 Calculation of automation scores for 32 trial farms

Farm number	Milking System	Milking-System-Score	Feeding System	Feeding System Score	Bedding System	Bedding System Score	Dung removal	Dung removal Score	Automation Score
1	AMS	2	CFS + Feed Pusher	1	manual	0	robot	2	**5**
2	AMS	2	CFS	0	manual	0	robot	2	**4**
3	AMS	2	CFS	0	manual	0	robot	2	**4**
4	CMS	0	AFS	2	manual	0	wheel loader	0	**2**
5	CMS	0	AFS	2	manual	0	slatted floors	0	**2**
6	CMS	0	AFS	2	manual	0	scraper	2	**4**
7	CMS	0	CFS	0	manual	0	scraper	2	**2**
8	AMS	2	AFS	2	manual	0	robot	2	**6**
9	CMS	0	CFS	0	manual	0	scraper	2	**2**
10	CMS	0	CFS	0	manual	0	scraper	2	**2**
11	AMS	2	CFS + Feed Pusher	1	manual	0	scraper	2	**5**
12	Batchmilking	1	AFS	2	automatic	2	scraper	2	**7**
13	AMS	2	AFS	2	manual	0	robot	2	**6**
14	AMS	2	AFS	2	automatic	2	scraper	2	**8**
15	AMS	2	AFS	2	manual	0	scraper	2	**6**
16	AMS	2	CFS + Feed Pusher	1	automatic	2	scraper	2	**7**
17	AMS	2	CFS + Feed Pusher	1	automatic	2	robot	2	**7**
18	AMS	2	CFS + Feed Pusher	1	automatic	2	robot	2	**7**
19	CMS	0	CFS	0	manual	0	wheel loader	0	**0**
20	AMS	2	AFS	2	manual	0	scraper	2	**6**
21	AMS	2	AFS	2	automatic	2	scraper	2	**8**
22	AMS	2	CFS + Feed Pusher	1	manual	0	slatted floors	0	**3**
23	CMS	0	CFS + Feed Pusher	1	manual	0	wheel loader	0	**1**
24	AMS	2	CFS + Feed Pusher	1	manual	0	scraper	2	**5**
25	AMS	2	CFS + Feed Pusher	1	manual	0	robot	2	**5**
26	AMS	2	AFS	2	manual	0	slatted floors	0	**4**
27	AMS	2	AFS	2	manual	0	robot	2	**6**
28	AMS	2	AFS	2	manual	0	robot	2	**6**
29	CMS	0	CFS	0	manual	0	scraper	2	**2**
30	AMS	2	CFS + Feed Pusher	1	manual	0	scraper	2	**5**
31	CMS	0	CFS	0	manual	0	slatted floors	0	**0**
32	CMS	0	CFS	0	manual	0	wheel loader	0	**0**
